# Tyrosine nitrations impaired intracellular trafficking of FSHR to the cell surface and FSH-induced Akt-FoxO3a signaling in human granulosa cells

**DOI:** 10.18632/aging.101964

**Published:** 2019-05-15

**Authors:** Ge Zhou, Rong-kui Hu, Gui-cheng Xia, Shi-hai Yan, Qing-ling Ren, Juan Zhao, Fei-hong Wang, Cheng-cai Huang, Qi Yao, Yong Tan, Ning-wei Zhao

**Affiliations:** 1 Department of Reproductive Medicine, Affiliated Hospital of Nanjing University of Chinese Medicine, Jiangsu Province Hospital of Chinese Medicine, Nanjing, China; 2 Laboratory of Pharmacology, Affiliated Hospital of Nanjing University of Chinese Medicine, Jiangsu Province Hospital of Chinese Medicine, Nanjing, China; 3 Department of Gynecology, Affiliated Hospital of Nanjing University of Chinese Medicine, Jiangsu Province Hospital of Chinese Medicine, Nanjing, China; 4 Shimadzu Biomedical Research Laboratory, Shanghai, China; 5 Department of Pathology and Pathophysiology, School of Medicine and Life Sciences, Nanjing University of Chinese Medicine, Nanjing, China; * Equal contribution

**Keywords:** poor ovarian response, FSHR, peroxynitrite, tyrosine nitrations, granulosa cells

## Abstract

Many infertile women suffered from poor ovarian response, and increased reactive oxygen species with age might mediate the poor ovarian response to FSH. In this study, we collected follicular fluids and isolated granulosa cells from female patients. Increased levels of peroxynitrite, tyrosine nitrations of FSH receptor (FSHR) and apoptosis were obviously detectable with decreased FSHR protein expressions in granulosa cells of the poor ovarian responders. In KGN (a human ovarian granulosa cell line) cells, exogenous peroxynitrite could sequester FSHR in the cytoplasm, and these dislocated FSHR might suffer from proteasome-mediated degradations. Here, we identified four peroxynitrite-mediated nitrated tyrosine residues of FSHR. Site-directed mutagenesis of FSHR revealed that Y626 was pivotal for intracellular trafficking of FSHR to the cell surface. Akt-induced inactivation of FoxO3a was required for the repression of FSH on granulosa cell apoptosis. However, peroxynitrite impaired FSH-induced Akt-FoxO3a signaling, while FSHR-Y626A mutant took similar effects. In addition, FoxO3a knockdown indeed impaired FSH-mediated cell survival, while FoxO3a-S253A mutant reversed that significantly.

## INTRODUCTION

Poor ovarian response (POR) is a pathological condition characterized by decreased follicular numbers and low E2 levels following controlled ovarian hyper-stimulation during in vitro fertilization (IVF) and embryo transfer surgeries. It has been reported that the incidence of POR was about 9%-24% among patients undergoing IVF [1]. Advanced age is one of the most remarkable risk factors for POR. The prevalence of POR increases with age, and in women over 40 years of age, it is over 50% [2]. In POR patients undergoing IVF, the success rate is extremely low and the total cancelled cycles are rather high due to the loss of follicles.

Nevertheless, the physiology of POR is not fully understood and the molecular events underlying POR remain unknown. Oxidative stress and mitochondrial dysfunction are among the most investigated possible mechanisms [3–6]. Mitochondria are the most abundant organelles in oocytes and early embryos that generate approximately 90% of reactive oxygen species (ROS) as the end products of oxygen metabolism, and then convert ROS into an inactive state via antioxidant defense mechanisms [7]. Ovarian aging may result from the requirements for more and more energy to maintain the functions of ovary, which is associated with the gradual reduction in the efficiency of repair processes during aging [8]. Alterations in energy metabolism can explain why the increased production of toxic ROS occurs, because the ROS eruption increased with age can seriously damage biomolecules and affect their normal functions. Oxidative stress could decrease FSH-stimulated granulosa cell (GC) steroid hormones, in particular E2, which is an important predictor of ovarian response [9]. Aldehyde dehydrogenase 3, member A2 is a ubiquitous nicotinamide adenine dinucleotide phosphate-dependent microsomal enzyme, which is involved in the detoxification of aldehydes generated by lipid peroxidation and its expression increases with the accumulation of ROS [10]. It was shown that ALDH3A2 expression in the GCs of IVF patients increased with age, which was negatively associated with FSHR expression and the number of total and mature oocytes obtained during ovarian stimulation [11]. As a G protein-coupled receptor (GPCR) consisting of intracellular, transmembrane and extracellular domains, FSHR is predominantly expressed in the ovarian GCs, which directly affects FSH-mediated biological effects [12]. Thus, increased ROS and diminished FSHR expression with age may explain the mechanism of POR.

Besides, GC apoptosis is associated with the increased oxidative stress, but the mechanism is still not clear now [13]. PI3K/Akt signaling has been identified as an important downstream pathway of FSH-mediated GC survival [14]. Protein kinase B (PKB)/Akt pathway is an essential pathway for cell survival and growth during development. This Akt-dependent survival function is mainly mediated by the FoxO family of transcription factors, which consists of FoxO1, 3a, 4, and 6 [15]. FoxOs also mediate cell cycle arrest, DNA repair and apoptosis [16]. The FoxO1 and FoxO4 are highly expressed in adipose tissue and skeletal muscle, respectively. FoxO6 is expressed predominantly in the developing and adult brain, while only FoxO3a is abundant in various tissues. Phosphorylation of FoxOs by Akt triggers the rapid relocalization of FoxOs from the nucleus to the cytoplasm. Akt phosphorylates FoxOs at three key regulatory sites (T32, S253, and S315 in the FoxO3a sequence) that are conserved from Caenorhabditis elegans to mammals and are part of a perfect consensus sequence for Akt phosphorylation [17]. Akt phosphorylation of FoxO3a could inactivate FoxO3a and inhibit cell apoptosis by suppressing the gene transcriptions of proapoptotic molecules, e.g., Bim and FasL [18]. It was previously reported that the repression of FSH on FoxO3a-driven gene expression of Bim was abolished by the PI3K inhibitor, and Bim induced porcine GC apoptosis during follicular atresia [19]. Thus, increased ROS may reverse FSH-mediated GC survival through Akt-FoxO3a signaling.

The aim of this study was to investigate the impact of oxidative stress on FSHR expressions in GCs from poor ovarian responders, and how the altered expressions of FSHR correlated with GC apoptosis.

## RESULTS

### Clinical characteristics of patients

The clinical characteristics of the POR and non-POR patients were shown in Supplementary Table 2. After comparing the POR group with the non-POR group, no statistical differences were found in terms of BMI. POR patients were a little older than non-POR patients, which was identical to the previous reports that the prevalence of POR increased with age. However, AMH levels were much lower in the POR group than in the non-POR group (p<0.05). As a product of the GCs, AMH envelop each egg and provide them energy, which also serve as a molecular biomarker for relative size of the ovarian reserve [20]. The FSH levels were markedly higher in the POR group than in the non-POR group (p<0.05). In females, FSH initiates follicular growth, specifically affecting GCs. By increasing aromatase expression, the FSH function in GCs is to stimulate the production of E2 [21]. Thus, as the FSH levels increases, the E2 levels increases accordingly. However, the E2 levels were surprisingly lower in the POR group than in the non-POR group (p<0.05), that aroused our great interest for the subsequent investigations.

### Molecular characteristics of human GCs from patients

**Figure 1:**
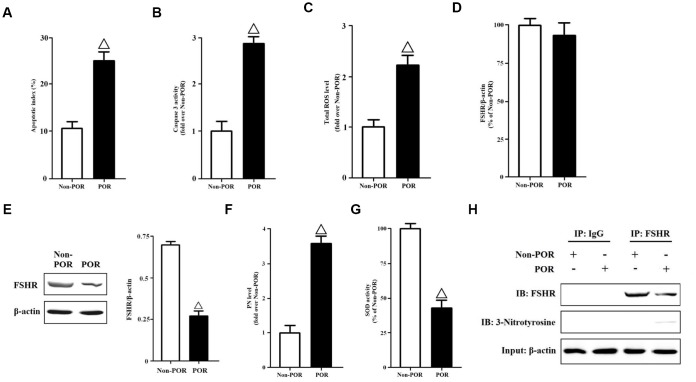
**Molecular characteristics of human GCs from non-POR and POR groups.** (**A**) The apoptotic indices of human GCs were measured using in Situ cell death detection kit. (**B**) The caspase-3 enzyme activities of human GCs were assayed using a commercial kit. (**C**) Total ROS levels of human GCs were measured by DCFH. (**D**) Relative mRNA expressions of FSHR in human GCs were determined by RT-PCR (β-actin as internal standard). (**E**) Relative protein expressions of FSHR in human GCs were determined by immunoblots (β-actin as internal standard). (**F**) PN levels of human GCs were measured by DHR. (**G**) The SOD activities of human GCs were measured using SOD activity assay kit. (**H**) Identification of tyrosine nitrated FSHR protein in human GCs. FSHR proteins were purified from cell lysates, and the relative protein expressions of nitrated FSHR were determined by immunoblots (β-actin as internal standard). Open triangle: p<0.05 vs. Non-POR group (n = 6–10).

We collected GCs from the FF of patients. The apoptotic indices were measured using in Situ cell death detection kit. The apoptosis rates of POR group were nearly three folds than those of non-POR group (Figure 1A). Meanwhile, the caspase-3 activities were detected by a commercial kit. The caspase-3 activities of non-POR group were obviously lower than those of POR group (Figure 1B). Due to the fact that increased ROS with age might mediate the POR to FSH during ovarian stimulation, total ROS levels of human GCs were measured here. As has been expected, total ROS levels were significantly higher in the POR group than in the non-POR group (Figure 1C). In order to explain why lower E2 levels occurred with higher FSH levels in the POR group, the gene and protein expressions of FSHR were measured in vivo. Surprisingly, no statistical differences were found in terms of FSHR mRNA levels (Figure 1D), while FSHR protein expressions were rather weaker in the POR group than in the non-POR group (Figure 1E), indicating that FSHR protein translation or stability might be hindered in the poor ovarian responders. Among all ROS, peroxynitrite (PN) is an oxidant and nitrating agent. Due to its oxidizing properties, PN can damage a wide array of molecules in cells, including DNA and proteins. Formation of PN requires free radical superoxide and nitric oxide [22, 23]. Thus, PN levels were measured here, and they were nearly four folds in the POR group than those in the non-POR group (Figure 1F). Superoxide dismutase (SOD) is responsible for scavenging the free radical superoxide in vivo [24]. After comparing the POR group with the non-POR group, the SOD activities were found to be significantly inhibited in the POR group (Figure 1G), which might contribute to the accumulation of PN.

### PN-mediated tyrosine nitration impaired the membrane anchoring of FSHR

**Figure 2:**
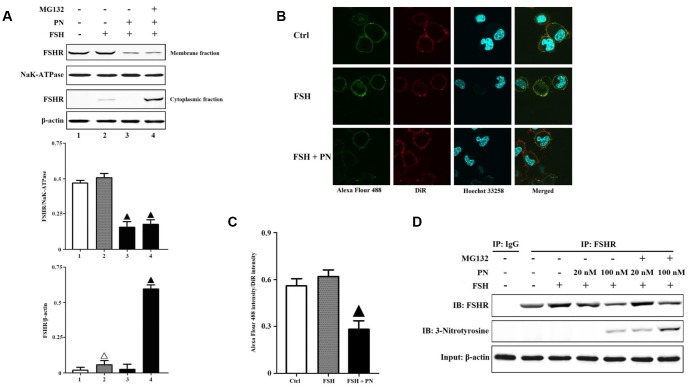
**PN-mediated tyrosine nitrations of FSHR abolished the membrane anchoring of FSHR and induced its degradation.** (**A**) PN impaired the membrane expression of FSHR and induced its degradation. KGN cells were incubated with or without PN (100 nM, 12 hrs), MG132 (30 μM, 4 hrs) followed by treatment with FSH (1 nM, 4 hrs). Relative protein expressions of FSHR in membrane fractions (NaK-ATPase as internal standard of membrane proteins) and cytoplasmic fractions (β-actin as internal standard of cytoplasmic proteins) were determined by immunoblots. (**B**) PN impaired the membrane anchoring of FSHR. KGN cells were incubated with or without PN (100 nM, 12 hrs), followed by treatment with FSH (1 nM, 4 hrs). Representative photo-micrographs of Alexa Flour 488 staining in KGN cells were examined by confocal microscopy, where Green fluorescence indicated Alexa Flour 488-positive FSHR proteins, red fluorescence indicated DiR-positive membrane, and blue fluorescence indicated Hoechst 33258-positive nuclei (Scale bar: 10 μm). (**C**) Relative ratios of FSHR to membrane were determined as the ratios of Alexa Flour 488 density to DiR intensity. (**D**) PN induced the protein nitrations of FSHR. KGN cells were incubated with or without PN (20 nM/100 nM, 12 hrs) and MG132 (30 μM, 4 hrs) followed by treatment with FSH (1 nM, 4 hrs). FSHR proteins were purified from cell lysates. The endogenous FSHR complex were analyzed by immunoblots (β-actin as internal standard). Open triangle: p<0.05 vs. Ctrl; Bold triangle: p<0.05 vs. FSH (n = 3–6).

As shown in Figure 1H, FSHR protein indeed suffered from greater tyrosine nitrations in the POR group than that in the non-POR group. Relative quantifications of FSHR proteins in human GCs of the POR patients revealed that NAC (a specific superoxide scavenger) treatment significantly increased the total expression of FSHR, especially the membrane expression of FSHR (Supplementary Figures 1–3). CHX (a specific protein synthesis inhibitor) treatment didn’t affect FSHR expression, indicating that FSHR protein translation wasn’t hindered in the poor ovarian responders. MG132 (a specific proteasome inhibitor) treatment also increased the total expression of FSHR, but it led to the accumulation of FSHR protein in the cytoplasm. These FSHR proteins which couldn't locate in the membrane correctly might suffer from proteasome-mediated degradations. The above findings suggested that PN-mediated tyrosine nitrations probably led to the protein degradations of FSHR. Then, we analyzed the KGN cells to explore PN-mediated effects on FSHR expression and function in vitro. The KGN cell line is considered as a very useful model for studying steroidogenesis, cell growth and FSHR-coupled signaling pathways in human GCs [25]. It was found that PN impaired the membrane expression of FSHR, while MG132 treatment prevented the degradations of cytoplasmic FSHR proteins (Figure 2A). The confocal imaging data also confirmed that PN-mediated tyrosine nitrations significantly impaired the membrane anchoring of FSHR proteins (Figure 2B, 2C). Meanwhile, PN indeed nitrated FSHR proteins dose-dependently (Figure 2D). FSH signals through FSHR, and stimulates GC survival via the activation of PI3K/Akt pathway [26]. Here, it was shown that FSH-mediated PI3K activities were indeed significantly reduced by PN dose-dependently (Supplementary Figure 4).

### Identification of nitrated tyrosine residues in FSHR protein

**Figure 3:**
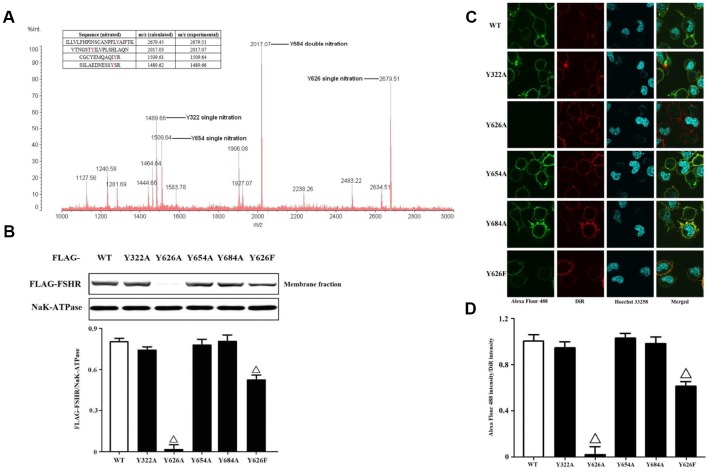
**Identification and functional analysis of nitrated tyrosine residues in FSHR protein.** (**A**) Identification of tyrosine nitrated sites in FSHR protein. The purified FSHR proteins from KGN cells were subject to MALDI-TOF MS analysis. Peaks with m/z 1489.66, m/z 1509.64, m/z 2017.07 and m/z 2679.51 corresponding to Y322 single nitration, Y654 single nitration, Y684 double nitration and Y626 single nitration were marked respectively. (**B**) Membrane expressions of FLAG-WT and its mutants. Relative protein expressions of FLAG-WT, Y322A, Y626A, Y654A, Y684A and Y626F in membrane fractions of KGN cells were determined by immunoblots (NaK-ATPase as internal standard of membrane proteins). (**C**) Representative photo-micrographs of Alexa Flour 488 staining in KGN cells transfected with FLAG-WT, Y322A, Y626A, Y654A, Y684A and Y626F. They were examined by confocal microscopy, where Green fluorescence indicated Alexa Flour 488-positive FSHR proteins, red fluorescence indicated DiR-positive membrane, and blue fluorescence indicated Hoechst 33258-positive nuclei (Scale bar: 10 μm). (**D**) Relative ratios of FLAG-WT and its mutants to membrane were determined as the ratios of Alexa Flour 488 density to DiR intensity. Open triangle: p<0.05 vs. FLAG-WT (n = 3–6).

FSHR functions can be regulated by PTMs, including glycosylation and phosphorylation [27]. The production of PN is capable of nitrating tyrosine residues in many proteins [28]. It is difficult to determine the exact sites of tyrosine nitrations before the coming of high resolution mass spectrometry. In order to identify the nitrated tyrosine residues in FSHR proteins, we adopted the immunoprecipitation coupled to MALDI-TOF analysis (Figure 3A). The data yielded the four nitrated tyrosine residues including Y322, Y626, Y654 and Y684, which were located in the extracellular N-terminus, Transmembrane domain 7 and cytoplasmic C-terminus, respectively. Meanwhile, we also quantified the tyrosine nitrated FSHR proteins (Y322/Y626/Y654/Y684 nitrated FSHR proteins) in human GCs of the POR patients (Supplementary Figure 5). NAC treatment markedly decreased the protein expression of tyrosine nitrated FSHR, and MG132 prevented the tyrosine nitrated FSHR proteins from proteasome-mediated degradations. Then, we mutated these tyrosine residues (Y322/Y626/Y654/Y684) to alanine and transfected them into KGN cells. It was found that Y322A, Y654A and Y684A of FSHR didn't affect the membrane expressions of FSHR at all (Figure 3B). Interestingly, the membrane expression of FSHR was almost disabled by FSHR-Y626A, indicating that Y626 residue was pivotal in the membrane anchoring of FSHR. Among all amino acids, phenylalanine shares the most similar structure with tyrosine, which also has an aromatic ring on its side chain. Therefore, we mutated Y626 to phenylalanine. As a result, FSHR-Y626F had the similar membrane anchoring to FSHR-WT, which was also identical to the confocal imaging data (Figure 3C, 3D).

### PN attenuated FSH-induced Akt-FoxO3a signaling

**Figure 4:**
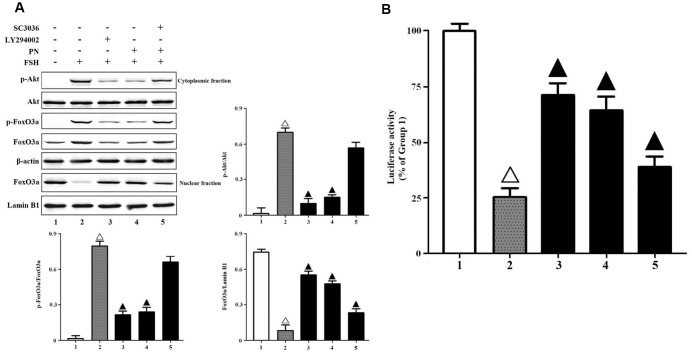
**PN attenuated Akt-FoxO3a signaling.** (**A**) PN attenuated Akt-FoxO3a activation and nuclear export of FoxO3a. KGN cells were incubated with or without PN (100 nM, 12 hrs), SC3036 (30 μM, 4 hrs) and LY294002 (30 μM, 4 hrs) followed by treatment with FSH (1 nM, 4 hrs). Relative protein expressions of p-Akt, Akt, p-FoxO3a, FoxO3a in cytoplasmic fractions (β-actin as internal standard) and FoxO3a in nuclear fractions (Lamin B1 as internal standard) were determined by immunoblots. (**B**) PN strengthened FoxO3a binding-dependent luciferase activities. KGN cells were transfected with the pGL3-Foxo3a consensus binding element-luciferase plasmids, and were incubated with or without PN (100 nM, 12 hrs), SC3036 (30 μM, 4 hrs) and LY294002 (30 μM, 4 hrs) followed by treatment with FSH (1 nM, 4 hrs). The luciferase activities were determined using the Dual-Luciferase Reporter Assay System. Open triangle: p<0.05 vs. Group 1; Bold triangle: p<0.05 vs. Group 2 (n = 3–6).

The production of PN may abolish FSH-induced Akt-FoxO3a signaling in GCs. Here, we found that PN significantly attenuated the phosphorylations of Akt and FoxO3a, and the nuclear export of FoxO3a, while LY294002 (a specific PI3K/Akt inhibitor) mimicked and SC3036 (a specific PI3K/Akt activator) reversed PN-mediated suppression of FSH-induced Akt-FoxO3a signaling in vitro (Figure 4A). A DNA fragment containing 8 copies of the sequence of Foxo3a consensus binding element (GTAAACA) was subcloned into the pGL3-luciferase reporter vector and transiently transfected into KGN cells as previously described [29]. FoxO3a binding-dependent luciferase activities were measured here. As a result, PN-mediated nuclear retention of FoxO3a significantly strengthened the transcriptional activities of FoxO3a, while LY29400 mimicked and SC3036 reversed those partially (Figure 4B). Next, we investigated whether tyrosine nitrations-induced FSHR protein instability alone could attenuate FSH-induced PI3K activities, because PN might nitrate not only FSHR but also other biomolecules. It was shown that FSHR-Y626A indeed impaired FSH-induced PI3K activities (Supplementary Figure 6). Meanwhile, FSHR-Y626A was able to attenuate the transcriptional activities of FoxO3a, and also upregulated the cell apoptosis and caspase-3 activities markedly (Supplementary Figures 7–9).

### FoxO3a inactivation was required for FSH-mediated cell survival

**Figure 5:**
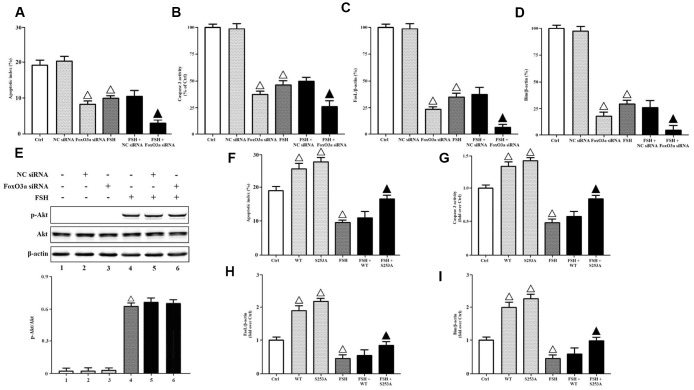
**FoxO3a inactivation was required for FSH-mediated cell survival. KGN cells transfected with or without NC siRNA or FSHR siRNA were incubated with or without FSH (1 nM, 4 hrs).** (**A**) The apoptotic indices were measured using in Situ cell death detection kit. (**B**) The caspase-3 enzyme activity was assayed using a commercial kit. Relative gene expressions of FasL (**C**) and Bim (**D**) were determined by RT-PCR (β-actin as internal standard). (**E**) Relative protein expressions of p-Akt and Akt were determined by immunoblots (β-actin as internal standard). On the other hand, KGN cells transfected with or without FoxO3a-WT and FoxO3a-S253A were incubated with or without FSH (1 nM, 4 hrs). (**F**) The apoptotic indices were measured using in Situ cell death detection kit. (**G**) The caspase-3 enzyme activity was assayed using a commercial kit. Relative gene expressions of FasL (**H**) and Bim (**I**) were determined by RT-PCR (β-actin as internal standard). Open triangle: p<0.05 vs. Ctrl; Bold triangle: p<0.05 vs. FSH (n = 3–6).

To determine whether FoxO3a inactivation was required for FSH-mediated cell survival, we transfected FoxO3a siRNA and NC siRNA into KGN cells, respectively. The apoptotic indices and caspase-3 activities were measured here. It was demonstrated that FoxO3a knockdown inhibited cell apoptosis and caspase-3 activities significantly, while FSH stimulation further aggravated FoxO3a silencing-mediated effects (Figure 5A, 5B). Besides, the gene expressions of FasL and Bim were also determined here. The data showed that FoxO3a knockdown downregulated the gene transcriptions of FasL and Bim significantly, while FSH stimulation further aggravated FoxO3a silencing-mediated effects (Figure 5C, 5D). Meanwhile, FoxO3a knockdown didn’t affect the Akt activation in response to FSH stimulation (Figure 5E), confirming that FoxO3a functioned as a downstream effector of PI3K/Akt. Next, we transfected KGN cells with FoxO3a-WT and FoxO3a-S253A respectively, where FoxO3a-S253A mimicked the FoxO3a hypo-phosphorylation. It was shown that FoxO3a overexpression increased cell apoptosis and caspase-3 activities without FSH stimulation, but it hardly affected those with FSH stimulation, while FoxO3a hypo-phosphorylation significantly reversed FSH-mediated cell survival (Figure 5F, 5G). Similarly, FoxO3a overexpression hardly affected the gene transcriptions of FasL and Bim with FSH stimulation, but it increased those without FSH stimulation, while FoxO3a hypo-phosphorylation significantly reversed FSH-mediated downregulation of FasL and Bim (Figure 5H, 5I).

## DISCUSSION

Management of female patients with POR is one of the major challenges in reproductive medicine. The primary causes of POR remain elusive and oxidative stress was proposed as one of the important contributors. In this study, our data yielded that higher FF FSH levels co-occurred with lower FF E2 levels in the POR group than in the non-POR group paradoxically, which might be due to the dysfunctions of GCs in the POR group. We isolated the GCs from the female patients, and found that increased cell apoptosis and intracellular PN occurred with decreased FSHR expression in the POR group.

Among all ROS, PN is a potent oxidative agent that is generated in vivo in mitochondria, whose formation requires free radical superoxide and nitric oxide. SOD can specifically scavenge free radical superoxide in vivo. We also showed that PN levels were negatively correlated with the SOD activities, suggesting that decreased activities of SOD might contribute to the increased PN levels with age. This in turn was reflected by the decreased activities of SOD, which further contributed to the oxidative damage. Our data was also coincided with the previous investigation which demonstrated that the expressions of SOD in female GCs diminished with age [30]. The PN was known to modify methionine, tryptophan, cysteine and tyrosine residues in proteins and peptides [31]. The widely known reaction of PN with biomolecules is the nitration of protein tyrosine residues to produce 3-nitrotyrosine [32]. The formation of protein 3-nitrotyrosine was originally addressed in early protein chemical studies with tetranitromethane (TNM) aimed at establishing the function of tyrosine residues in proteins [33]. This now-established PTM attracts considerable interest to biomedical research, because it can alter protein function, which is associated with acute and chronic disease states and can be a predictor of disease risk [34]. Thus, PN levels were measured in this study. The data showed that the higher PN levels were significantly associated with the POR. Palumbo A, et al previously reported that the addition of PN induced a 2.7-fold reduction in the protein expressions of FSHR in vitro [35]. However, the authors didn't further investigate how PN affected FSHR expression. Here, we firstly reported that PN-mediated tyrosine nitrations of FSHR were much more in the POR group than in the non-POR group, when gene transcriptions of FSHR were hardly affected. Next, we found that PN dose-dependently caused the tyrosine nitrations and proteasome-mediated degradations of FSHR in vitro. In addition, it was demonstrated that PN attenuated FSH-mediated PI3K activities.

**Figure 6:**
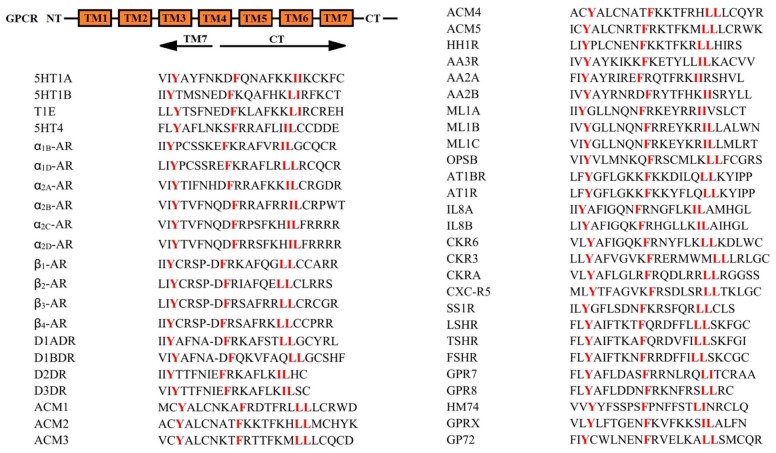
**The conserved Y(X)_6_F(X)_6_LL motif in the CTs of G protein coupled receptors.** The data were constructed from the alignments described in the G protein-coupled receptor database: information system for G protein-coupled receptors (www.gpcrdb.org). Human sequences are shown when available. 5HT1A, human 5-hydroxytryptamine 1A receptor; 5HT1B, human 5-hydroxytryptamine 1B receptor; 5HT1E, human 5-hydroxytryptamine 1E receptor; 5HT4, human 5-hydroxytryptamine 4 receptor; α_1B_-AR, human α_1B_ adrenergic receptor; α_1D_-AR, human α_1D_ adrenergic receptor; α_2A_-AR, human α_2A_ adrenergic receptor; α_2B_-AR, rat α_2B_ adrenergic receptor; α_2C_-AR, human α_2C_ adrenergic receptor; α_2D_-AR, human α_2D_ adrenergic receptor; β_1_-AR, human β_1_ adrenergic receptor; β_2_-AR, human β_2_ adrenergic receptor; β_3_-AR, human β_3_ adrenergic receptor; β_4_-AR, human β_4_ adrenergic receptor; D1ADR, human dopamine D(1A) receptor; D1BDR, human dopamine D(1B) receptor; D2DR, human dopamine D (2) receptor; D3DR, human dopamine D (3) receptor; ACM1, human muscarinic acetylcholine receptor M1; ACM2, human muscarinic acetylcholine receptor M1; ACM3, human muscarinic acetylcholine receptor M1; ACM4, human muscarinic acetylcholine receptor M1; ACM5, human muscarinic acetylcholine receptor M1; HH1R, human histamine H1 receptor; AA3R, human adenosine A3 receptor; AA2A, human adenosine A2a receptor; AA2B, human adenosine A2b receptor; ML1A, human melatonin type 1A receptor; ML1B, human melatonin type 1B receptor; ML1C, chicken melatonin type 1C receptor; OPSB, chicken blue-sensitive opsin; AT1BR, human angiotensin II type-1B receptor; AT1R, rat angiotensin II type-1A receptor; IL8A, human interleukin-8 receptor A; IL8B, human interleukin-8 receptor B; CKR6, human C-C chemokine receptor type 6; CKR3, human C-C chemokine receptor type 3; CKRA, human C-C chemokine receptor type 10; CXC-R5, human C-X-C chemokine receptor type 5; SS1R, human somatostatin receptor type 1; LSHR, human lutropin-choriogonadotropic hormone receptor precursor; TSHR, human thyrotropin receptor precursor; FSHR, human follicle stimulating hormone receptor precursor; GPR7, human neuropeptides B/W receptor type 1; GPR8, human neuropeptides B/W receptor type 2; HM74, human probable GPCR HM74; GPRX, mouse probable GPCR GPR33; GP72, mouse probable GPCR GPR72 precursor. NT: N-terminus; TM1-7: Transmembrane domain 1-7; CT: Carboxyl terminus.

Tyrosine nitration is a stable, covalent modification. A single tyrosine nitration results in the mass increase of 45 Da in the tyrosine residue, while a double nitration results in the mass increase of 90 Da in the tyrosine residue. Both singly and doubly nitrated tyrosine residues are stable under MS/MS condition (collision-induced dissociation) [36, 37]. With the help of high resolution MALDI-TOF, we identified four nitrated tyrosine residues of FSHR protein in vitro and ex vivo. Meanwhile, the site-directed mutagenesis and confocal imaging data in KGN cells firstly revealed that Y626 was pivotal for the membrane expression of FSHR, because Y626A mutation of FSHR nearly lost the membrane anchoring capacity and Y626F had the similar cell surface expression to FSHR-WT. Since we found that LSHR, TSHR and FSHR shared a similar motif for their export from the ER to the cell surface, we searched the GPCR database to see if this motif was conserved in the superfamily of GPCR. As shown in Figure 6, the sequence Y(X)_6_F(X)_6_LL (where X represents any amino acid residues and L is leucine or isoleucine) is highly conserved in the membrane-proximal CTs of many GPCR. The highly conserved F(X)_6_LL motif required for receptor transport to the cell surface was previously clarified [38, 39]. Since Y626 is located in the proximal region of the F(X)_6_LL motif, we speculated that Y(X)_6_F(X)_6_LL sequence of FSHR was extremely important in its ability to couple to G proteins as well as its trafficking to the cell surface [40]. The residues, Y626, F633 and L640/L641 with a strictly constrained spatial relationship may provide a specific interactive site mediating the interaction of FSHR with selective proteins to facilitate their transport from the ER to the membrane lipid raft. The Y(X)_6_F(X)_6_LL motif may also provide a docking site for machinery proteins involved in receptor folding to the state competent for ER export.

In response to growth factors, FoxO3a are phosphorylated by Akt, resulting in their nuclear export and cytoplasmic sequestration that interferes with their transcriptional activities and thus promotes cell proliferation [41]. Several reports demonstrated that the promoters of a number of proapoptotic genes such as Bim and FasL, contained a consensus FoxO3a binding element and the transcriptions of these genes would be induced by FoxO3a [42, 43]. Here, FoxO3a silencing indeed inhibited KGN cell apoptosis with or without FSH treatment significantly, while FoxO3a overexpression upregulated that without FSH treatment. Unexpectedly, FoxO3a overexpression hardly affected FSH-mediated cell survival, which might be due to that recombinantly expressed FoxO3a proteins were mainly sequestered into the cytoplasm by Akt. However, FoxO3a hypo-phosphorylation, whose nuclear entry was weakened, markedly reversed FSH-mediated cell survival. These data really indicated that FoxO3a was required for FSH-mediated cell survival. Although Akt-dependent cell growth and survival can be regulated by multiple signaling pathways [44], Akt-induced inactivation of FoxO3a was required for the repression of FSH on GC apoptosis.

**Figure 7:**
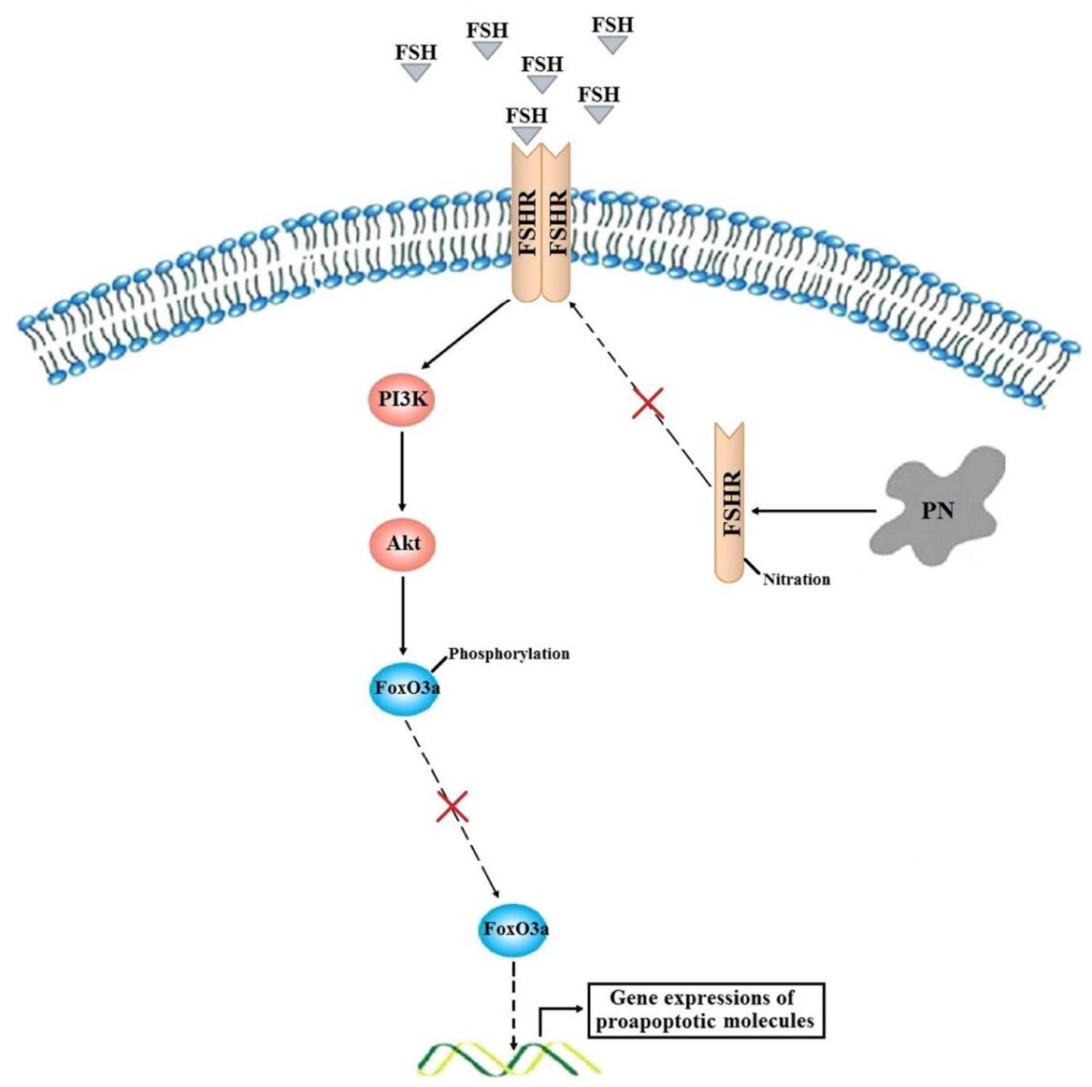
**A proposed model of how PN increases FoxO3a-mediated apoptosis of human GCs in the poor ovarian responders.**

In conclusion, a model is thus proposed in which FSH signals through FSHR and activate PI3K/Akt signaling cascade. This study demonstrated that accumulation of PN prevented the membrane location of FSHR and the subsequent Akt-dependent phosphorylations of FoxO3a, thereby disabling FoxO3a from nuclear exporting. Then, the non-phosphorylated FoxO3a stayed within the nucleus, and initiated the gene transcriptions of proapoptotic molecules, finally resulting in increased apoptosis of GCs (Figure 7). Considering that PN-mediated tyrosine nitrations leading to cytoplasmic retention and degradation of FSHR might be implicated in the POR, further developments of therapeutic strategies targeting oxidative stress in GCs would take effects.

## MATERIALS AND METHODS

### Patients

In this study, we collected follicular fluid samples from 40 female patients undergoing IVF. All patients were recruited from Department of Reproductive Medicine, Affiliated Hospital of Nanjing University of Chinese Medicine, between April 2016 and April 2016. 20 patients with POR were diagnosed according to the Bologna Criteria. The ages of all patients ranged from 30 to 40. In order to define the presence of POR, at least two of the following three features had to be confirmed: (i) advanced maternal age or any other risk factor for POR; (ii) previous POR; or (iii) an abnormal ovarian reserve test. Two POR episodes after maximal stimulation were sufficient to define a patient as a poor responder in the absence of advanced maternal age or an abnormal ovarian reserve test. The other 20 patients were non-POR with normal antral follicle counts whose infertility was caused by tubal or male infertility factors. All patients recruited had regular menstrual cycles (26-35 days). We excluded patients suffering from other related diseases, such as polycystic ovary syndrome, Turner syndrome, thyrotoxicosis, hyperprolactinemia or recurrent spontaneous abortion, as well as patients who had undergone ovarian surgery or chemotherapy. In addition, several mutations can affect FSHR’s biological activity, and have been linked to primary ovarian failure, infertility, and so on [45]. The A189V mutation of FSHR gene results in a complete blocking of FSH action and failure of human chorionic gonadotropin (hCG) to increase ovarian secretion of E2 [46]. In order to avoid the interference of FSHR gene variants, we excluded patients having specific gene mutations of FSHR during our sample collections. All patients underwent controlled ovarian hyper-stimulation protocols with different FSH /human menopausal gonadotropin starting doses depending on their age. This study was approved by the Human Care and Use Committee of Affiliated Hospital of Nanjing University of Chinese Medicine and written consent was obtained from each patient included in this study.

### Collection of human FF and GCs

During IVF, patients received human chorionic gonadotrophin when the diameter of their follicles was bigger than 18 mm. Oocytes were collected 36 hrs after human chorionic gonadotrophin injection by transvaginal ultrasound-guided puncture and aspiration of follicles with a diameter of 18 to 20 mm. The FF (2 ml) from the first aspirated follicle, not contaminated by blood, was carefully collected from each donor, centrifuged at 2000 g for 20 mins, and stored at 80°C. Meanwhile, GCs from the same patients undergoing IVF cycles were collected using the isolation protocol described previously [47].

### Reagents and antibodies

PN was purchased from Cayman Chemical. MG132 (one specific proteasome inhibitor) and NAC (N-acetyl-cysteine, one specific superoxide scavenger) were obtained from Sigma Aldrich. SC3036 (one specific PI3K/Akt agonist) was purchased from Santa Cruz Biotechnology. LY294002 (one specific PI3K/Akt inhibitor) was obtained from Cell Signaling Technology. FSH (human pituitary) was purchased from Merck Millipore. FSHR pAbs (orb213952) were obtained from Biorbyt. β-actin pAbs (YT0099), Lamin B1 pAbs (YT5180), p-Akt (T308) pAbs (YP0590) and Akt pAbs (YT0173) were purchased from Immunoway. NaK-ATPase mAbs (ab76020), 3-Nitrotyrosine mAbs (ab52309), control rabbit IgG (ab172730), Flag-tag pAbs (ab122902), p-FoxO3a (S253) mAbs (ab154786), FoxO3a mAbs (ab53287), Alexa-flour 488-conjugated secondary Abs (ab150077) and Cycloheximide (CHX, one specific protein synthesis inhibitor, ab120093) were obtained from Abcam.

### RT-PCR assay

Total RNA was extracted with Trizol reagent (Gibco) as described by the manufacturer. RT-PCR was performed using the Access RT-PCR Introductory System (Promega) with indicated primers (Supplementary Table 1). The threshold count values were normalized to beta-actin to calculate the fold change in expression.

### Measurements of total ROS, peroxynitrite (PN) and SOD activity

Total ROS and PN levels were measured as previously described [48, 49]. The cells were incubated with 20 μM DCFH-DA (Sigma Aldrich) for 45 mins to determine total ROS level, or 50 μM DHR (Sigma Aldrich) for 30 mins to determine PN level. The fluorescent products (λ_ex_ = 485/λ_em_ = 520 nm after the reaction of DCFH with total ROS; λ_ex_ = 500/λ_em_ = 536 nm after the reaction of DHR with PN were analyzed by a fluorescence spectrometer (RF-5301PC, Shimadzu). The activities of SOD (Superoxide dismutase) were measured using SOD activity assay kit (Abcam) following the instructions of the manufacturer.

### Apoptotic index and caspase-3 activity assays

Cells were cultured on poly-L-lysine-coated Labtek II chamber slides and treated as indicated below. The slides were washed once with PBS, fixed for 40 mins with 4% paraformaldehyde, washed twice with PBS, permeabilized with 0.2% Triton X-100 for 5 mins, and washed twice with PBS. Apoptotic index was measured using an in Situ cell death detection kit (Roche) following the instructions of the manufacturer. Apoptotic cells were detected under a fluorescence microscope (Olympus IX81), when the apoptotic nuclei were identified by HRP-conjugated anti-fluorescein antibodies. Caspase-3 enzyme activity was studied by using a caspase-3 colorimetric activity assay kit (Sigma Aldrich) following the instructions of the manufacturer.

### Immunoprecipitation and immunoblotting

The intracellular proteins were extracted using NE-PER Cytoplasmic/Nuclear Extraction Kit or Mem-PER Plus Membrane Protein Extraction Kit according to the manufacturer’s protocol (Thermo Fisher Scientific). The total protein concentration of tissue supernatant was determined using a Bio-Rad Protein Assay (Bio-Rad Laboratories). Proteins were immunoprecipitated with indicated antibodies respectively [50]. The precleared Protein A/G Plus-agarose beads (Santa Cruz Biotechnology) were incubated with immunocomplexes for 2h and washed four times with the lysis buffer. The immunoprecipitates were subjected to SDS-PAGE followed by transferring onto nitrocellulose membranes (Hybond-C, Amersham Biosciences). The membranes were incubated overnight with the appropriate primary antibodies. The bound antibodies were then visualized using HRP-conjugated secondary antibodies. The intensities of the bands were quantified with BandScan.

### Cell culture

The KGN cell line (human granulosa-like tumor cell line) was gifted from Nanjing University. KGN cells were seeded in culture dishes and cultured in the DMEM/F12 medium supplemented with 10% fetal bovine serum (Thermo Fisher Scientific), penicillin (100 U/ml) and streptomycin (100 μg/ml) in a humidified incubator containing 5% CO_2_ at 37°C.

### Identification of nitrated tyrosine residues in FSHR protein

KGN cells were incubated with PN (100 nM, 12 hrs) and MG132 (30 μM, 4 hrs) followed by treatment with FSH (1 nM, 4 hrs). The cell lysates were subject to SDS-PAGE. FSHR bands were excised manually from CBB stained gels, destained with 100 mM Ammonium bicarbonate/50% Acetonitrile and dehydrated in Acetonitrile by vacuum drying. The dehydrated gel slices were subject to trypsin digestion. Tryptic digested peptides were extracted 50% Acetonitrile/0.1% trifluoroacetic acid (TFA). The extracted peptides were lyophilized and reconstituted in 0.1% TFA and desalted using MonoTip C18 Columns (Shimadzu GL). The desalted peptides were subject to MALDI-TOF MS analysis as previously described [51]. This raw data acquired by MALDI-7090 (Shimadzu Kratos) was then searched against SwissProt Database with possible modifications of single and double nitration on tyrosine (+45 Da for single nitration and +90 Da for double nitration) using MALDI Solutions following the instructions of the manufacturer [52].

### Site-directed mutagenesis and transfection

FLAG-tagged FSHR plasmid and HA-tagged FoxO3a plasmid were obtained from Nanjing Newtop Biotechnology Co., Ltd. FSHR mutations (Tyr to Ala or Phe) of Y322A, Y626A, Y654A, Y684A, Y626F and FoxO3a (Ser to Ala) mutation of S253A were generated by Fast Mutagenesis System (TransGen Biotech) and verified by DNA sequencing (Invitrogen). Transient transfections of KGN cells were performed using LipofectAMINE 2000 (Invitrogen) according to the manufacturer's instructions.

### Immunofluorescence microscopy

Cells were rinsed two times with PBS followed by 4% paraformaldehyde fixation for 30 mins and permeabilizing with 0.2% Triton X-100 for 20 mins. After being blocked by 5% BSA to reduce non-specific binding, the cells were incubated with anti-FLAG pAbs overnight. After washing (0.1% Tween-20 in PBS), cells were incubated with Alexa Flour 488-conjugated secondary Abs for 1 hr. DiR near-IR membrane probe (Abcam) was used to show the cell membranes, and Hoechst 33258 (Sigma Aldirch) was used to show the nuclei. Slides were mounted and examined under a fluorescence microscope (Olympus IX81).

### RNA interference study

The pSuperior.retro.puro vector (OligoEngine) was used for the expression of siRNA in KGN cells. FoxO3a siRNA vector was generated by a gene-specific insert (5′-GTGGAGCTGGACCCGGAGT-3′) to target FoxO3a. A negative control (NC) vector was constructed using an insert (5′-GTGTCTGTAGGAGTCATCC-3′) with no significant homology to any mammalian gene sequence. Transfection was performed using LipofectAMINE 2000, according to the manufacturer’s protocol. Briefly, KGN cells were transfected with 10 nM FoxO3a siRNA or NC siRNA for 48 hrs, followed by further analysis. FoxO3a levels in KGN cells were assessed by immunoblots after transient transfection. NC siRNA was used to assess non-specific gene-silencing effects.

### Statistics

All data are presented as means ± SEM and were analyzed using two-tailed Student's t-test between two groups. p < 0.05 was considered statistically significant. All statistical analyses were performed using GraphPad Prism 5 software (GraphPad).

## Supplementary Material

Supplementary File
