# Supplementary material for: Tyrosine nitrations impaired intracellular trafficking of FSHR to the cell surface and FSH-induced Akt-FoxO3a signaling in human granulosa cells
Source: Aging (Albany NY). 2019 May 15;11(10):3094–116. doi: 10.18632/aging.101964 (PMC6555443; doi:10.18632/aging.101964)
Supplement: Supplementary File [file aging-11-101964-s001.pdf]

SUPPLEMENTARY MATERIALS

Supplementary Information

Relative quantifications of FSHR proteins in human GCs of the POR patients

GCs of the POR patients were collected and cultured in DMEM/F12 media supplemented with 10% fetal calf serum, 1% pyruvate (PAA laboratories), 1% penicillin/streptomycin (Gibco) and 0.1% biotine/pan-tothenate mixture (Sigma-Aldrich) in a humidified atmosphere of 20% O_2 and 5% CO_2 at 37°C. The intracellular proteins were extracted from GCs using NE-PER Cytoplasmic/Nuclear Extraction Kit or Mem-PER Plus Membrane Protein Extraction Kit according to the manufacturer's protocol (Thermo Fisher Scientific). The total protein con-centration of tissue supernatant was determined using a Bio-Rad Protein Assay (Bio-Rad Laboratories). Proteins were immunoprecipitated with anti-FSHR antibodies (orb213952, Biorbyt) or control rabbit IgG (ab172730, Abcam), respectively. The precleared Protein A/G Plus-agarose beads (Santa Cruz Biotechnology) were incubated with immuno-complexes for 2 hrs and washed four times with the lysis buffer to remove non-specifically bound proteins. The precipitate samples obtained in immunoprecipitation experiment were reduced with 10 mM DTT for 1 hr, followed by alkylation with 40 mM iodoacetamide for 1 hr under dark conditions. Samples were diluted with 50 mM ammonium bicarbonate to a less than 1 M final urea concentration before protein digestion with trypsin (Promega) at a mass ratio of 1:20 (trypsin: protein) overnight at 37°C. Following tryptic digestion, peptide samples were desalted using MonoTip C18 (Shimadzu Biotech). The eluted peptides were dried in a SpeedVac and then labeled with iTRAQ reagents (Thermo Fisher Scientific) as previously described [1]. Each of the four samples (Control, CHX, CHX + NAC, and CHX + MG132) was labeled with four vials of iTRAQ isobaric reagent (114, 116, 119 and 121), respectively. After incubation for 2 hrs at room temperature, the reaction was stopped by acidification with formic acid (1%). The four iTRAQ-labeled peptide samples were then combined and desalted using MonoTip C18 (Shimadzu Biotech). The eluted peptides were dried in a SpeedVac and resuspended in 0.1% formic acid prior to LC-MALDI analysis. The prepared peptides were separated by nano-HPLC (Prominence HPLC, Shimadzu Biotech). Briefly, a 5 µl aliquot of the peptides was loaded directly onto the column (300 µm i.d. x 15 cm, 3 µm, Pepmap C18 (LC Packings)) and separated using a two stage linear gradient: (A = 5% acetonitrile + 0.1% formic acid, B = 80/20 acetonitrile + 0.1% formic acid). The eluents passed through a UV/Vis detector (220 nm) and was mixed with MALDI matrix (CHCA; 5 mg/ml in 50/50 acetonitrile/0.1% TFA) were automatically deposited onto MALDI target plates by the LC spotting system (AccuSpot, Shimadzu Biotech). These spotted samples were automatically analyzed by MALDI-TOF/TOF MS (MALDI-7090, Shimadzu Kratos). The peptide mass fingerprints and peptide ion MS/MS spectra were acquired automatically on MALDI-7090. The total MS/MS data was searched against SwissProt Database using the following parameters: trypsin digestion allowing up to 1 missed cleavages, fixed modifications of cysteine (carbamidomethylation) and iTRAQ (lysine and N-termini), variable modifications of methionine (oxidation) and tyrosine (single nitration or double nitration), precursor peptide tolerance of 0.2 Da, and MS/MS tolerance of 0.8 Da. Search results with e values less than 0.01 were judged as positive identifications. Relative ratios of FSHR peptides were quantified by Proteome Discoverer Software (Thermo Fisher Scientific) via the quantification of iTRAQ labeled peptides as previously described [2]. To minimize contaminating near isobaric ions, only the peptides with isolation specificity more than 75% were quantified. For redundant peptides, the relative ratio was calculated from the pair with the highest summed reporter ion intensity. The relative ratios of FSHR peptides were used as the basis for the calculation of the mean value for FSHR proteins across all three biological replicates. An unpaired t-test was used to determine whether changes in peptide abundances were significant.

PI3K activity assay

PI3K activity was evaluated using K1000s PI3-Kinase Activity ELISA kit (Echelon Biosciences) and human purified PI3Kα from cell lysates [3]. PI3K reactions are run with the Class I PI3K physiological substrate PIP2. The enzyme reactions, PIP3 standards, and controls are then mixed and incubated with PIP3 binding protein that is highly specific and sensitive to PIP3. This mixture is then transferred to a PIP3-coated microplate for competitive binding. Afterwards, a peroxidase-linked secondary detector and colorimetric detection is used to detect the amount of PIP3 produced by PI3K through comparing -the enzyme reactions with a PIP3 standard curve.

Supplementary References

1. Montgomery H, Rustogi N, Hadjisavvas A, Tanaka K, Kyriacou K, Sutton CW. Proteomic profiling of breast tissue collagens and site-specific characterization of hydroxyproline residues of collagen alpha-1-(I). J Proteome Res. 2012; 11(12):5890–902. https://doi.org/10.1021/pr300656r PMID:23110299

2. Polisetty RV, Gautam P, Sharma R, Harsha HC, Nair SC, Gupta MK, Uppin MS, Challa S, Puligopu AK, Ankathi P, Purohit AK, Chandak GR, Pandey A, Sirdeshmukh R. LC-MS/MS analysis of differentially expressed glioblastoma membrane proteome reveals altered calcium signaling and other protein groups of regulatory functions. Mol Cell Proteomics. 2012; 11(6):M111.013565. https://doi.org/10.1074/mcp.M111.013565 PMID:22219345

3. Furtado CM, Marcondes MC, Carvalho RS, Sola-Penna M, Zancan P. Phosphatidylinositol-3-kinase as a putative target for anticancer action of clotrimazole. Int J Biochem Cell Biol. 2015; 62:132–41. https://doi.org/10.1016/j.biocel.2015.03.004 PMID:25794423

Supplementary Figures

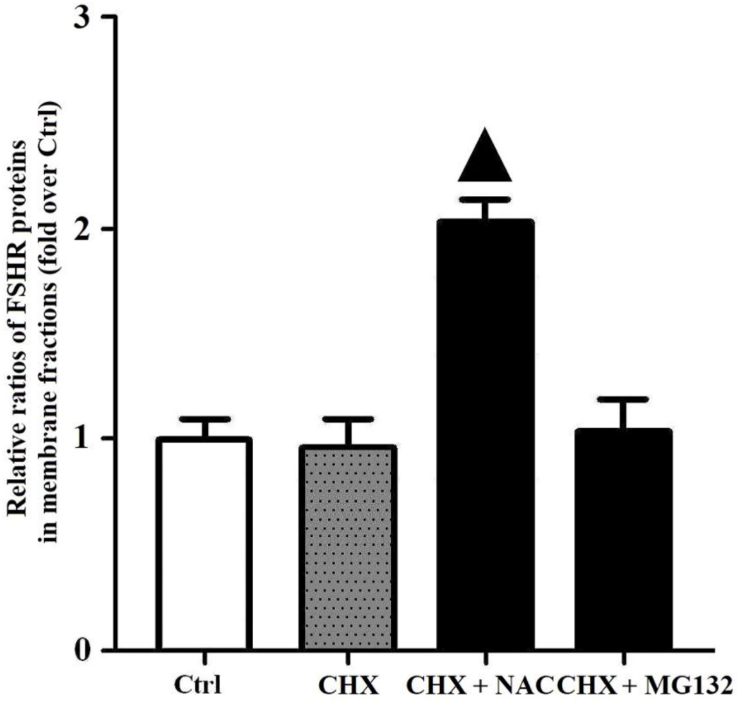

Supplementary Figure 1. Relative quantifications of FSHR proteins in membrane fractions of human GCs. Human GCs of the POR patients were incubated with or without NAC (0.3 mM, 12 hrs), MG132 (30 μM, 4 hrs) followed by treatment with CHX (20 μM, 4 hrs). Relative ratios of FSHR proteins in membrane fractions were measured by iTRAQ-based LC-MALDI analysis. Open triangle: $p<0.05$ vs. Ctrl; Bold triangle: $p<0.05$ vs. CHX (n = 3–6).

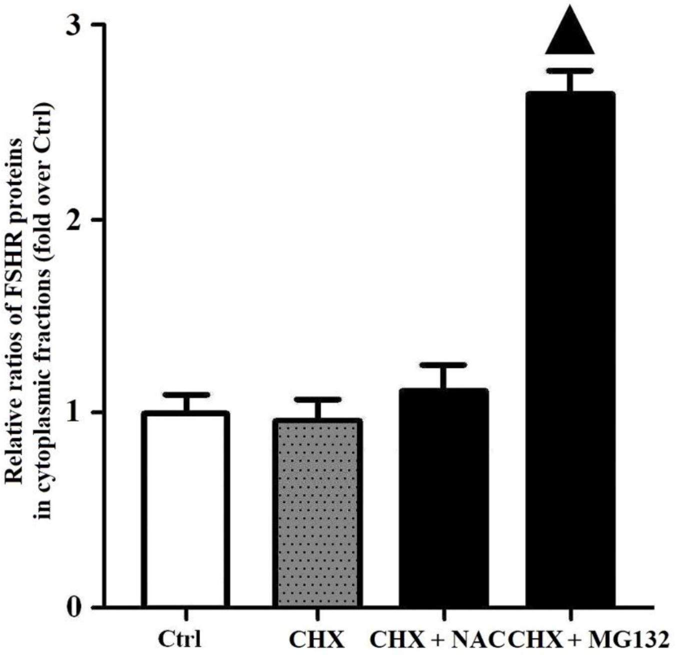

Supplementary Figure 2. Relative quantifications of FSHR proteins in cytoplasmic fractions of human GCs. Human GCs of the POR patients were incubated with or without NAC (0.3 mM, 12 hrs), MG132 (30 μM, 4 hrs) followed by treatment with CHX (20 μM, 4 hrs). Relative ratios of FSHR proteins in cytoplasmic fractions were measured by iTRAQ-based LC-MALDI analysis. Open triangle: $p<0.05$ vs. Ctrl; Bold triangle: $p<0.05$ vs. CHX (n = 3–6).

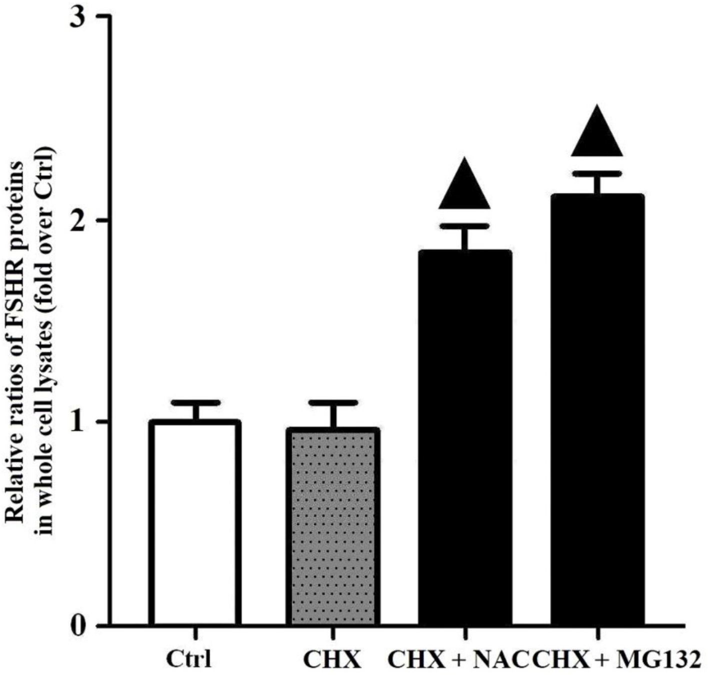

Supplementary Figure 3. Relative quantifications of FSHR proteins of human GCs. Human GCs of the POR patients were incubated with or without NAC (0.3 mM, 12 hrs), MG132 (30 μM, 4 hrs) followed by treatment with CHX (20 μM, 4 hrs). Relative ratios of FSHR proteins in whole cell lysates were measured by iTRAQ-based LC-MALDI analysis. Open triangle: $p<0.05$ vs. Ctrl; Bold triangle: $p<0.05$ vs. CHX (n = 3–6).

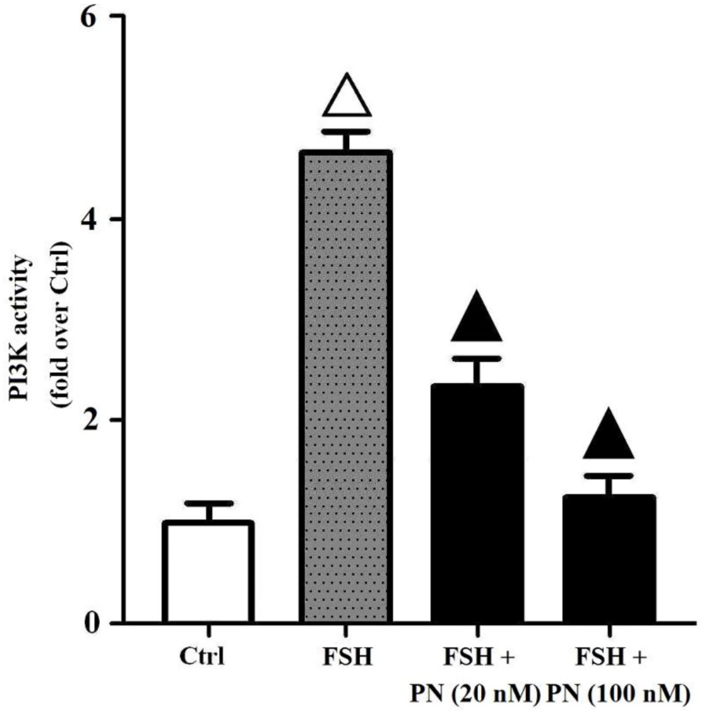

Supplementary Figure 4. PN downregulated the PI3K activities. KGN cells were incubated with or without PN (20 nM/100 nM, 12 hrs) followed by treatment with FSH (1 nM, 4 hrs). The PI3K activities were evaluated using K1000s PI3-Kinase Activity ELISA kit. Open triangle: p<0.05 vs. Ctrl; Bold triangle: p<0.05 vs. FSH (n = 3–6).

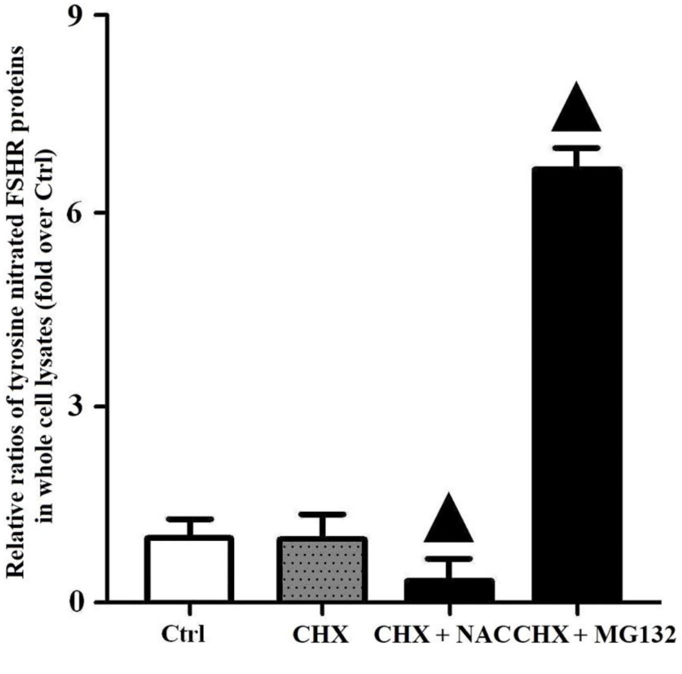

Supplementary Figure 5. Relative quantifications of Tyrosine nitrated FSHR proteins of human GCs. Human GCs of the POR patients were incubated with or without NAC (0.3 mM, 12 hrs), MG132 (30 μM, 4 hrs) followed by treatment with CHX (20 μM, 4 hrs). Relative ratios of tyrosine nitrated FSHR proteins (Y322/Y626/Y654/Y684 nitrated FSHR proteins) in whole cell lysates were measured by iTRAQ-based LC-MALDI analysis. Open triangle: p<0.05 vs. Ctrl; Bold triangle: p<0.05 vs. CHX (n = 3–6).

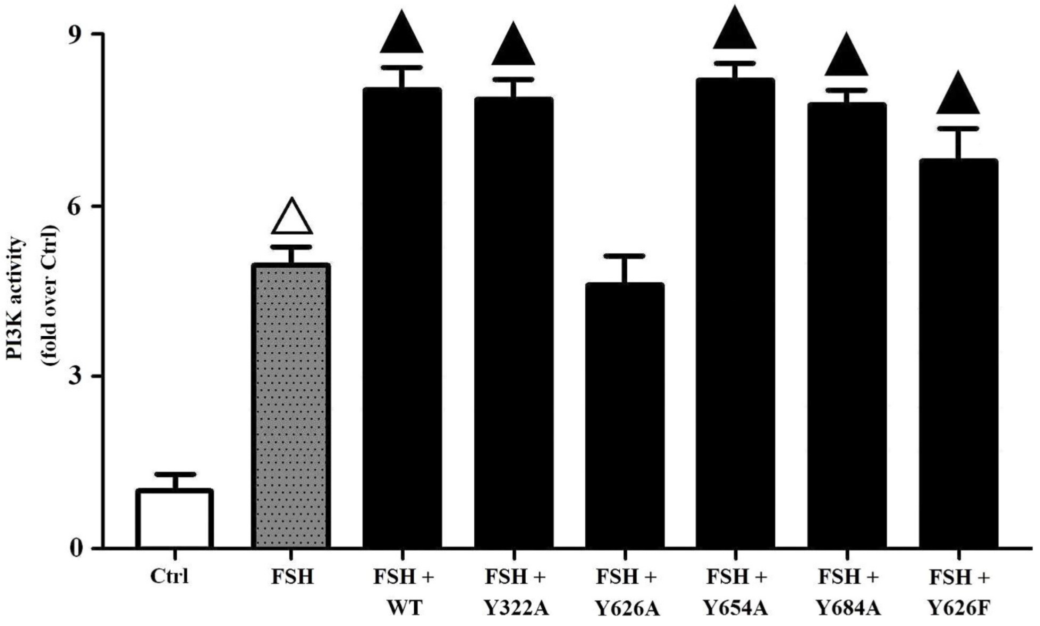

Supplementary Figure 6. PI3K activities of FLAG-WT and its mutants. The PI3K activities of KGN cells transfected with or without FLAG-WT, Y322A, Y626A, Y654A, Y684A and Y626F followed by treatment with FSH (1 nM, 4 hrs) were evaluated using K1000s PI3-Kinase Activity ELISA kit. Open triangle: $p<0.05$ vs. Ctrl; Bold triangle: $p<0.05$ vs. FSH (n = 3–6).

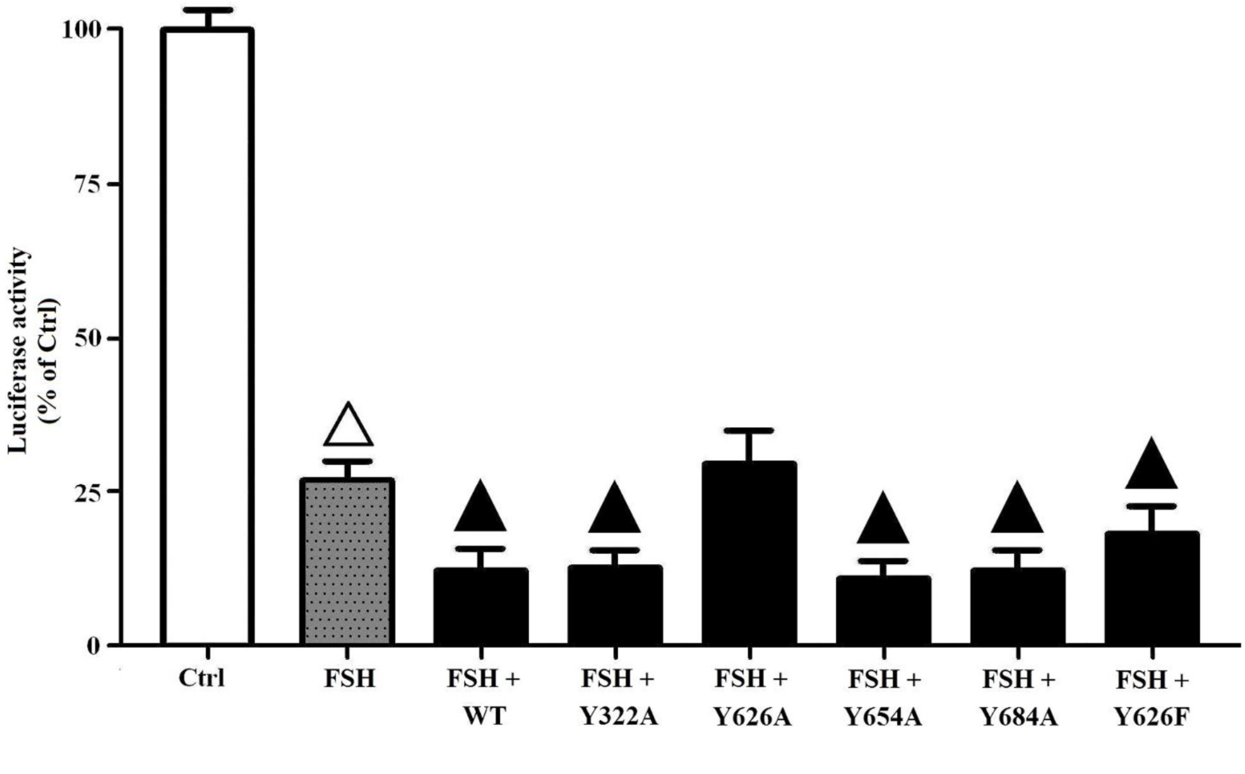

Supplementary Figure 7. FoxO3a binding-dependent luciferase activities of FLAG-WT and its mutants. The KGN cells transfected with the pGL3-Foxo3a consensus binding element-luciferase plasmids were then transfected with or without FLAG- WT, Y322A, Y626A, Y654A, Y684A and Y626F followed by treatment with FSH (1 nM, 4 hrs). The luciferase activity was determined using the Dual-Luciferase Reporter Assay System. Open triangle: $p<0.05$ vs. Ctrl; Bold triangle: $p<0.05$ vs. FSH (n = 3–6).

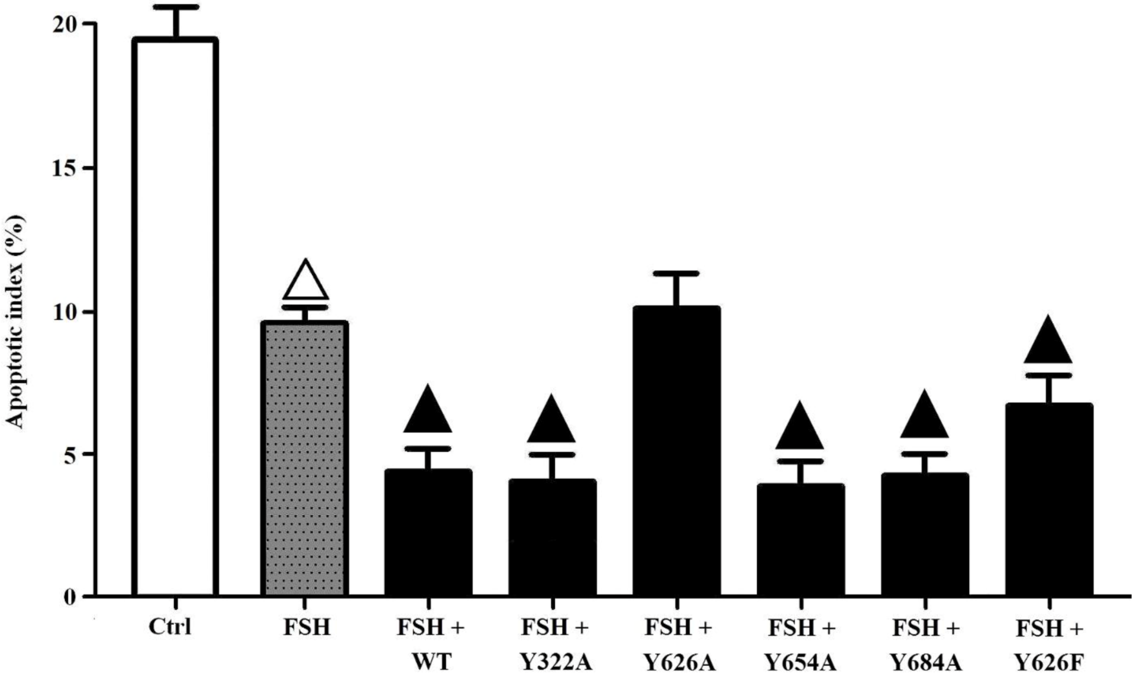

Supplementary Figure 8. Apoptotic indices of FLAG-WT and its mutants. The apoptotic indices of KGN cells transfected with or without FLAG-WT, Y322A, Y626A, Y654A, Y684A and Y626F followed by treatment with FSH (1 nM, 4 hrs) were measured using in Situ cell death detection kit. Open triangle: $p<0.05$ vs. Ctrl; Bold triangle: $p<0.05$ vs. FSH (n = 3–6).

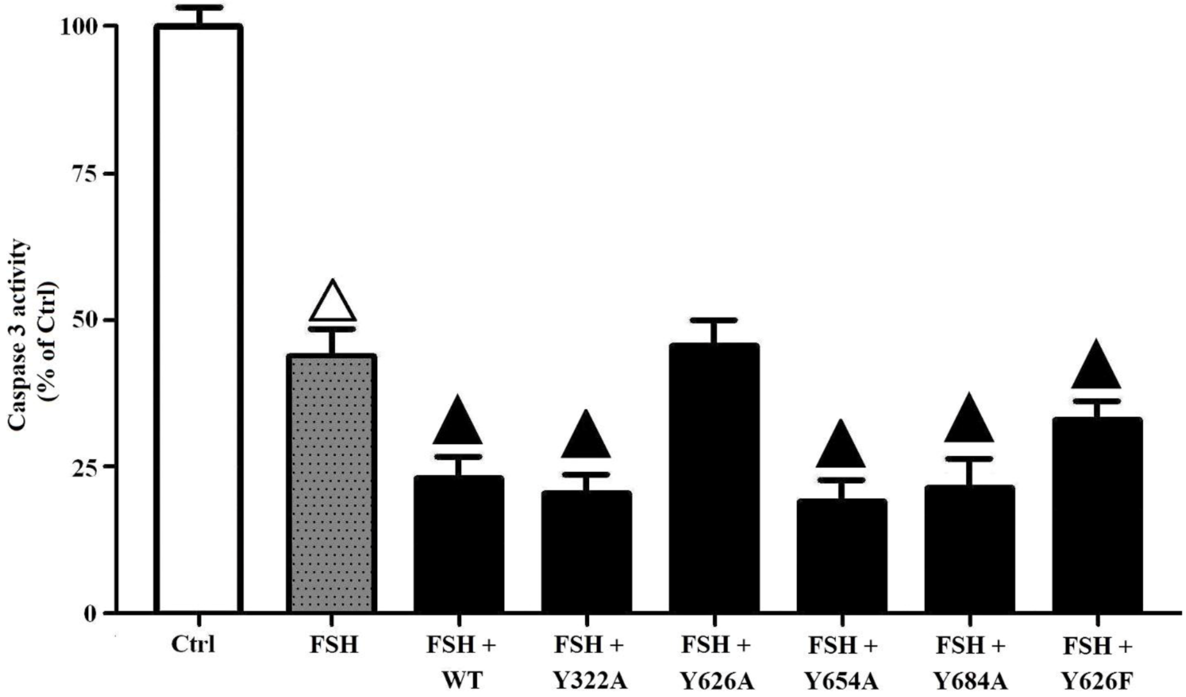

Supplementary Figure 9. Caspase-3 activities of FLAG-WT and its mutants. The caspase-3 enzyme activities of KGN cells transfected with or without FLAG-WT, Y322A, Y626A, Y654A, Y684A and Y626F followed by treatment with FSH (1 nM, 4 hrs) were assayed using a commercial kit. Open triangle: $p<0.05$ vs. Ctrl; Bold triangle: $p<0.05$ vs. FSH (n = 3–6).

Supplementary Tables

Supplementary Table 1. Primers list.

Gene	Primer pair 5′-3′)
**FSHR**	Fw: TTCCTTACTGCCAACTCTCC
	Rw: TCATACACCAGACCGTCTGA
**Bim**	Fw: CTACCAGATCCCCACTTTTC
	Rw: GCCCTCCTCGTGTAAGTCTC
**FasL**	Fw: TCCAACTCAAGGTCCATGCC
	Rw: CAGAGAGAGCTCAGATACGTTT
**β-actin**	Fw: TTCCAGCCTTCCTTCCTGG
	Rw: TTGCGCTCAGGAGGAGCAAT

Supplementary Table 2. Clinical characteristics of female patients from non-POR and POR groups.

Group	Age (yrs)	BMI	AMH (ng/ml)	FSH baseline (IU/L)	E2 baseline (pg/ml)
**Non-POR**	31.76±0.48	20.44±0.12	0.71±0.14	8.06±0.88	50.84±15.27
**POR**	37.17±0.63	20.81±0.98	3.18±0.37	11.19±1.33	36.75±12.15

Abbreviations: POR: poor ovarian response group; Non-POR: non poor ovarian response group; BMI: body mass index; AMH: anti mullerian hormone; FSH: follicle-stimulating hormone; E2: estradiol.
